# Low dose 100 cGy irradiation as a potential therapy for pulmonary hypertension

**DOI:** 10.1002/jcp.28723

**Published:** 2019-04-22

**Authors:** Pamela C. Egan, Olin D. Liang, Laura R. Goldberg, Jason M. Aliotta, Mandy Pereira, Theodor Borgovan, Mark Dooner, Giovanni Camussi, James R. Klinger, Peter J. Quesenberry

**Affiliations:** ^1^ Division of Hematology/Oncology, Department of Medicine Warren Alpert Medical School of Brown University Providence Rhode Island; ^2^ Division of Pulmonary, Critical Care and Sleep Medicine, Department of Medicine, Rhode Island Hospital Warren Alpert Medical School of Brown University Providence Rhode Island; ^3^ Department of Medical Sciences University of Torino Torino Italy

**Keywords:** endothelial progenitor cells, low dose irradiation, pulmonary hypertension

## Abstract

Pulmonary hypertension (PH) is an incurable disease characterized by pulmonary vascular remodeling and ultimately death. Two rodent models of PH include treatment with monocrotaline or exposure to a vascular endothelial growth factor receptor inhibitor and hypoxia. Studies in these models indicated that damaged lung cells evolve extracellular vesicles which induce production of progenitors that travel back to the lung and induce PH. A study in patients with pulmonary myelofibrosis and PH indicated that 100 cGy lung irradiation could remit both diseases. Previous studies indicated that murine progenitors were radiosensitive at very low doses, suggesting that 100 cGy treatment of mice with induced PH might be an effective PH therapy. Our hypothesis is that the elimination of the PH‐inducing marrow cells by low dose irradiation would remove the cellular influences creating PH. Here we show that low dose whole‐body irradiation can both prevent and reverse established PH in both rodent models of PH.

## 1 INTRODUCTION

The World Health Organization (WHO) defines five categories of pulmonary hypertension (PH), many of which are highly lethal and not very responsive to existent therapies. The therapy of PH has been a robust area of research. Major therapies for PH include endothelin receptor antagonists, phosphodiesterase‐5 inhibitors, soluble guanylate cyclase stimulators, and prostacyclin pathway agents. These have shown short‐term improvements, but there are questions as to their long‐term efficacy and none are curative. New agents continue to be studied, including anti‐inflammatory and antiproliferative drugs. All approaches have variable side effects (Badlam & Bull, 2017; Mishra, Singh, & Kaluski, 2017). Steensma, Hook, Stafford, and Tefferi (2002) have reported impressive responses of four patients with myelofibrosis and myeloid metaplasia involving the lung with severe PH, treated with 100 centigray (cGy) lung radiation. The myelofibrosis and the PH resolved. These remissions lasted from 6 to 12 months. A follow up of 100 cGy lung irradiation in 57 patients with myelofibrosis and extramedullary hematopoiesis in the lungs showed responses of the lung myelofibrosis in 30 days with a median time to response of 10 days (1–174 days) and in 20 patients followed for over 1 year no apparent toxicity related to the lung irradiation was seen. “In the group with concurrent active cardiac or pulmonary conditions 15 patients had clinical improvement” after irradiation (Chaudhry, Merrell, Tefferi, & Neben, 2015). These studies suggest a potential role for either lung or WBI as a therapy for PH.

Studying monocrotaline (MCT)‐induced PH in mice, we recently reported marrow‐derived endothelial progenitors from the MCT‐treated mice could induce PH in irradiated normal mice (Aliotta et al., 2017). Many other studies have suggested a role for bone marrow‐derived cells in the pathogenesis of pulmonary arterial hypertension. Farha et al. (2011) reported that nonaffected family members of patients with familial pulmonary arterial hypertension displayed elevated circulating levels of CD34^+^CD133^+^ progenitor cells, which were comparable with their affected relatives with pulmonary arterial hypertension, and had a significant increase in marrow fibrosis compared with healthy unrelated controls. These investigators hypothesized that a subclinical myeloproliferative process may be intrinsic to the development of pulmonary arterial hypertension. It was also observed that in the plexiform lesions and perivascular spaces of remodeled pulmonary arteries in patients with pulmonary arterial hypertension there were c‐kit‐positive cells (Montani et al., 2011). This hypothesis was further supported by a study in which mice transplanted with bone marrow‐derived CD133 progenitor cells from patients with pulmonary arterial hypertension developed PH (Asosingh et al., 2012). Recently, a genetic model of PH using the Bmpr2 mutant mice was published (Yan et al., 2015). They found that mutant bone marrow cells caused PH, with remodeling and inflammation, when transplanted into control mice, whereas control bone marrow cells had a protective effect against the development of disease when transplanted into mutant mice.

We had previously studied the sensitivity of marrow progenitor/stem cells to low dose gamma irradiation. We found that 100 cGy whole‐body irradiation (WBI) was profoundly stem cell toxic but minimally myelotoxic (Stewart et al., 2001). Further, there is published data indicating that human endothelial stem cells are quite radiosensitive (Mendonca et al., 2011). In addition, in clinical trials treating cancer patients we demonstrated that 100 cGy WBI was well tolerated and without apparent long‐term toxicities (Colvin et al., 2009). Based on the above, we hypothesized that low dose WBI would selectively eliminate disease‐causing endothelial progenitors in the murine model of PH without inducing significant toxicity. We tested our hypothesis in both the Sugen/hypoxia (Su/Hx)‐induced PH and MCT‐induced PH mouse models (Aliotta et al., 2013, 2015; Vitali et al., 2014).

## 2 MATERIALS AND METHODS

### 2.1 Su/Hx‐induced and MCT‐induced PH mouse models

The Su/Hx‐PH protocol consisted of 3 weekly subcutaneous injections of the Sugen vascular endothelial growth factor receptor‐2 inhibitor SU5416 (Tocris) at 20 mg/kg in 100 μl dimethyl sulfoxide or vehicle alone. During the 3‐week SU5416 treatment, mice were exposed to hypoxia (8.5% O_2_) or normoxia (Nx) for 3 weeks. For the MCT‐induced PH mouse model, cohorts of mice received weekly subcutaneous injections of MCT (60 mg/kg; Sigma) resuspended in 100 μl of saline or 100 μl of saline only (vehicle) for 4 weeks. Development of PH was determined by measurement of the right ventricular systolic pressure (RVSP) and right ventricular (RV) hypertrophy (i.e., Fulton's index). To measure RVSP, mice were anesthetized via intraperitoneal injection with ketamine (100 mg/kg) and xylazine (10 mg/kg). RVSP was measured through a trans‐thoracic route with a Millar catheter transducer PVR‐1030 (ADInstruments Inc., Colorado Springs, CO) and data were collected and analyzed using the LabChart software v8.1.3 (ADInstruments). To access RV hypertrophy, whole hearts were weighted after the atria and great vessels were trimmed. The right ventricles were then dissected away from the heart and left ventricle plus septum (LV + S) were weighted. Fulton's index (RV/LV + S) was then calculated.

### 2.2 Immunohistochemistry

To demonstrate muscularization of the distal pulmonary arteriole vessel wall, immunostaining was performed using a rabbit polyclonal antibody against α‐smooth muscle actin (α‐SMA; 1:100; ab5694; Abcam, Cambridge, MA). Slides were then incubated with the EnVision + Dual Link System‐HRP solution (Agilent Technologies, Santa Clara, CA) containing anti‐rabbit immunoglobulins conjugated to a peroxidase‐labeled polymer. Following chromogenic development, the slides were counterstained with hematoxylin. Images of α‐SMA staining were then taken by using a Nikon Eclipse E800 microscope (Nikon Instruments Inc., Melville, NY) equipped with a camera and SPOT Advanced 4.7 software (Diagnostic Instruments Inc., Sterling Heights, MI).

### 2.3 Low dose 100 cGy irradiation

Low dose 100 cGy irradiation was conducted by using a Gammacell® 40 Exactor (Best Theratronics LTD., Ottawa, ON, Canada) with a 137 Cesium source.

### 2.4 Statistical analysis

All analysis was performed using the GraphPAD Prism software. All values are expressed as mean ± standard error of the mean. Comparison between groups was assessed using one‐way analysis of variance with multiple comparisons. A value of *p* < 0.05 was considered significant.

## 3 RESULTS AND DISCUSSION

**Figure 1:**
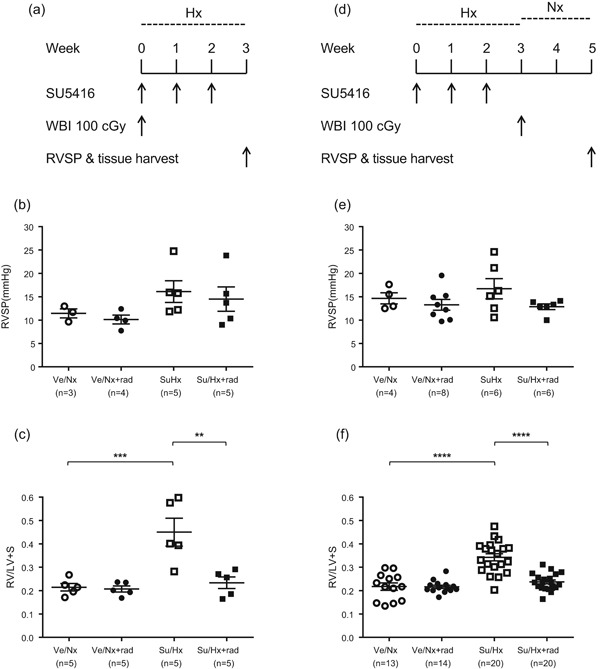
Low dose 100 cGy WBI can prevent PH development as well as reverse established PH in Su/Hx‐induced PH mouse model. (a) Schematic of experimental design using 100 cGy WBI to prevent PH development in a prophylactic model. Mice underwent 100 cGy WBI and then 3 weeks of hypoxia with weekly Sugen 5416 injections. (b) Low dose 100 cGy WBI appears to have reduced RVSP in irradiated compared to nonirradiated Su/Hx‐PH mice in the prophylactic model. (c) Low dose 100 cGy WBI prevents the development of right ventricular hypertrophy in Su/Hx‐treated mice as indicated by Fulton's Index (RV/LV + S) in the prophylactic model. (d) Schematic of experimental design using 100 cGy WBI to reverse established PH in a therapeutic model. Mice underwent 3 weeks of hypoxia treatment with weekly Sugen 5416 injections then received 100 cGy WBI. (e) Low dose 100 cGy WBI appears to normalize RVSP in irradiated compared to nonirradiated Su/Hx‐PH mice in the therapeutic model. (f) Fulton's Index indicates that 100 cGy WBI reverses established right ventricular hypertrophy in Su/Hx‐treated mice in the therapeutic model. Data in (b), (c), (e), and (f) are mean ± standard error of the mean, **p < 0.01, ***p < 0.001, and ****p < 0.0001, one‐way analysis of variance with multiple comparisons. LV + S: left ventricle plus septum; PH: pulmonary hypertension; RV: right ventricular; RVSP: right ventricular systolic pressure; Su/Hx: Sugen 5416‐ and hypoxia‐treated mice; Su/Hx + rad: Sugen 5416‐, hypoxia‐, and radiation‐treated mice; Ve/Nx: vehicle‐ and normoxia‐treated mice; Ve/Nx + rad: vehicle‐, normoxia‐, and radiation‐treated mice; WBI: whole‐body irradiation

**Figure 2:**
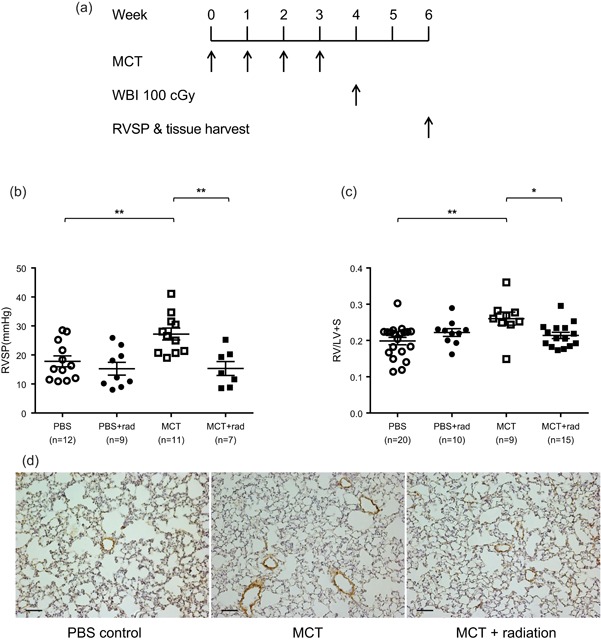
Low dose 100 cGy WBI can reverse established PH in MCT‐induced PH mouse model. (a) Schematic of experimental design using 100 cGy WBI to reverse established PH in MCT‐induced PH model. Mice received weekly MCT injection for 4 weeks before receiving 100 cGy WBI. (b) Low dose 100 cGy WBI reverses RVSP in MCT‐PH mice. (c) Low dose 100 cGy WBI reverses right ventricular hypertrophy in MCT‐treated mice as indicated by Fulton's index (RV/LV + S). (d) Immunohistochemical staining of α‐SMA (brown color) in the lung from PBS control mice, MCT‐treated mice, MCT and radiation‐treated mice. Original magnification ×200. Data in (b) and (c) are mean ± standard error of the mean, *p < 0.05 and **p < 0.01, one‐way analysis of variance with multiple comparisons. LV + S: left ventricle plus septum; MCT: monocrotaline; PH: pulmonary hypertension; RV: right ventricular; RVSP: right ventricular systolic pressure; PBS: phosphate‐buffered saline‐treated mice; PBS + rad: PBS‐ and radiation‐treated mice; MCT: MCT‐treated mice; MCT + rad: MCT‐ and radiation‐treated mice; WBI: whole‐body irradiation [Color figure can be viewed at wileyonlinelibrary.com]

In a prophylactic model where mice underwent WBI before initiation of Su/Hx treatment (Figure 1a), the mice that received irradiation (Su/Hx + rad) had an improvement in RVSP (Figure 1b), and they did not develop RV hypertrophy compared to nonirradiated Su/Hx mice (Figure 1c). In a therapeutic model, mice were exposed to 100 cGy WBI after the induction of PH (Figure 1d). The mice that underwent WBI had a normal RVSP (Figure 1e), and they had a resolution of RV hypertrophy compared to nonirradiated Su/Hx mice (Figure 1f). These results indicated that the Su/Hx‐induced PH had been reversed by 100 cGy WBI. The pulmonary vascular remodeling studies did not show increased thickness in the Su/Hx‐treated mice, which is consistent with other studies in mice where vascular pruning or drop out occurred and vascular thickness while present 1 week after the last Sugen injection was not present after 10 weeks of normoxia (Vitali et al., 2014). In our studies the mean wall thickness to diameter ratios (wt/d) in the irradiated and the nonirradiated Su/Hx mice was comparable. This was not significantly different from wall thicknesses in the vehicle control mice with or without irradiation (Data not shown). We also performed immunohistochemical (IHC) staining of α‐SMA to assess the muscularization of distal pulmonary arterioles (≤50 μm). However, the IHC staining showed no significant difference between the irradiated and the nonirradiated Su/Hx‐PH mice, since both of which had minimal staining of α‐SMA in the pulmonary arterioles (data not shown). Next, we tested our hypothesis in the MCT‐induced PH model (Figure 2a). As shown in Figure 2b and c, low dose 100 cGy WBI significantly reversed both the RVSP and RV hypertrophy. IHC staining of α‐SMA also suggested that low dose 100 cGy WBI reversed muscularization of distal pulmonary arterioles (≤50 μm; Figure 2d).

Our current study shows that low dose WBI at 100 cGy, a level which has previously been established as minimally myelotoxic, can prevent the development of PH as well as reverse established PH in two different mouse models of PH. Our previous work (Aliotta et al., 2017) showing that endothelial progenitors from mice with MCT‐induced PH were a cellular cause of PH, along with the known radiosensitivity of this class of cells (Mendonca et al., 2011), suggests that eliminating this class of cells was responsible for reversal of the disease. Given the previously established minimal toxicity of 100 cGy to the hematopoietic system of mice (Stewart et al., 2001) and clinical studies in humans showing that this level of irradiation is also minimally myelotoxic (Colvin et al., 2009), this approach offers a potential therapy for human PH which may be effective and nontoxic. Whether this might be effective for primary pulmonary arterial hypertension or one of the other 4 WHO classifications of PH remains an open question. The potential relationship of PH to myeloid disorders, specifically myelofibrosis and myeloid metaplasia, was mentioned above and further suggests that low dose radiation may be an interesting up‐front therapy for myelofibrosis and myeloid metaplasia.

Low dose irradiation was also shown to prevent the development of PH in Su/Hx‐treated mice. When 100 cGy WBI was administered before the initiation of the Su/Hx regimen, the development of RV hypertrophy was prevented. This raises the possibility of using low dose irradiation in situations, such as sickle cell anemia, where the development of PH is a major risk factor (Gladwin, 2016). While the study reported by Steensma utilized selective lung irradiation (Steensma et al., 2002), we used WBI. We rationalized that if marrow‐derived endothelial progenitors were responsible for the development of PH; the WBI was more likely to prevent continued seeding of toxic marrow cells to the lung. Clearly, it will be of interest to investigate the impact of isolated lung irradiation along with the duration of reversal of the disease.

Overall it would appear that low dose irradiation depletes the PH inducing marrow‐derived progenitors thus both preventing and reversing the induced PH. This assumption is further backed by two other therapy models in which modulation of toxic marrow progenitors appears to be effective for preventing or reversing rodent PH. We have previously reported that marrow‐derived mesenchymal stem cell (MSC) vesicles can effectively remit PH in the rodent models (Aliotta et al., 2013). If these MSC vesicles are incubated with PH‐inducing progenitors these progenitors no longer induce PH. Alternate data indicates that marrow endothelium in adults induces PH‐inducing progenitors in the PH rodent models. The endothelial to hematopoietic transition appears to be dependent upon the transcription factor RUNX1. Inhibition of RUNX1 is also capable of remitting PH by inhibiting the endothelial to hematopoietic transition (Liang et al., 2017). These data further suggest that PH, at least in some instances, may be a marrow myeloid progenitor disorder. Thus, there appear to be three different approaches which modulate PH‐inducing marrow progenitors which can treat PH: MSC‐derived vesicles, RUNX1 inhibition, and low dose nonmyelotoxic WBI.

## CONFLICT OF INTERESTS

The authors declare that there are no conflict of interests.

## AUTHOR CONTRIBUTIONS

P. C. E. and O. D. L.: collection and/or assembly of data, data analysis and interpretation, manuscript writing; L. R. G., J. M. A., M. P., T. B., M. D., G. C., and J. R. K.: collection and/or assembly of data, data analysis and interpretation; O. D. L. and P. J. Q.: conception and design, collection and/or assembly of data, data analysis and interpretation, manuscript writing, and final approval of manuscript. All the authors have read and approved the manuscript.
